# Bayesian spatio-temporal modeling of severe acute respiratory syndrome in Brazil: A comparative analysis across pre-, during, and post-COVID-19 eras

**DOI:** 10.1016/j.idm.2024.12.010

**Published:** 2024-12-19

**Authors:** Rodrigo de Souza Bulhões, Jonatha Sousa Pimentel, Paulo Canas Rodrigues

**Affiliations:** a Department of Statistics, IME, Federal University of Bahia, Salvador, BA, Brazil; b Department of Statistical Methods, IM, Federal University of Rio de Janeiro, Rio de Janeiro, RJ, Brazil; c Department of Statistics, CCEN, Federal University of Pernambuco, Recife, PE, Brazil; d Econometrics and Business Statistics, Monash University, Australia

**Keywords:** Spatio-temporal generalized linear model for areal unit data, Bayesian spatio-temporal modeling, Severe acute respiratory syndrome, COVID-19, Brazilian health regions

## Abstract

This paper presents an investigation into the spatio-temporal dynamics of Severe Acute Respiratory Syndrome (SARS) across the diverse health regions of Brazil from 2016 to 2024. Leveraging extensive datasets that include SARS cases, climate data, hospitalization records, and COVID-19 vaccination information, our study employs a Bayesian spatio-temporal generalized linear model to capture the intricate dependencies inherent in the dataset. The analysis reveals significant variations in the incidence of SARS cases over time, particularly during and between the distinct eras of pre-COVID-19, during, and post-COVID-19. Our modeling approach accommodates explanatory variables such as humidity, temperature, and COVID-19 vaccine doses, providing a comprehensive understanding of the factors influencing SARS dynamics. Our modeling revealed unique temporal trends in SARS cases for each region, resembling neighborhood patterns. Low temperature and high humidity were linked to decreased cases, while in the COVID-19 era, temperature and vaccination coverage played significant roles. The findings contribute valuable insights into the spatial and temporal patterns of SARS in Brazil, offering a foundation for targeted public health interventions and preparedness strategies.

## 1 Introduction

Severe Acute Respiratory Syndrome (SARS) is a highly contagious respiratory illness that emerged as a global health concern in the early 21st century (Peiris, Yuen, et al., 2003). Characterized by severe respiratory distress and a propensity for rapid transmission, SARS can be caused by the influenza virus and other etiological agents, such as respiratory syncytial virus, parainfluenza, and adenovirus (Araujo et al., 2020). The hallmark symptoms include fever, cough, and difficulty breathing, often leading to pneumonia in severe cases (Peiris, Lai, et al., 2003). The virus primarily spreads through respiratory droplets, making person-to-person transmission a significant challenge for public health officials. The world witnessed the first major outbreak of SARS in 2002–2003, which originated in China (Peiris et al., 2004) and affected various countries across Asia, North America, and Europe (Curley y Thomas, 2004). Stringent public health measures, international collaboration, and robust research efforts ultimately controlled the outbreak.

However, COVID-19, caused by the novel coronavirus SARS-CoV-2, emerged in 2019 as a profound global health crisis, affecting millions of individuals worldwide. Characterized by respiratory symptoms ranging from mild to severe, and in some cases leading to acute respiratory distress syndrome, COVID-19 has challenged healthcare systems, economies, and societal norms (Acter et al., 2020; El Zowalaty y Järhult, 2020). Its relationship with SARS lies in their shared viral origins. Coronaviruses cause both diseases, with SARS caused by SARS-CoV and COVID-19 by SARS-CoV-2. In Brazil, the impact of COVID-19 has been substantial, with varying regional dynamics and a complex interplay of social, economic, and healthcare factors influencing the spread and management of the disease (Sott et al., 2022). The country has faced challenges related to healthcare capacity, testing infrastructure, and the implementation of public health measures (Kameda et al., 2021).

The prevalence and incidence of Severe Acute Respiratory Syndrome (SARS) in Brazil have been dynamic, reflecting the evolving nature of the disease over time. The Brazilian territory is divided into five geographical regions and 456 health regions, different from the smaller Brazilian administrative regions, the 5570 municipalities. Due to its magnitude and diversity, surveillance data has indicated varying rates of SARS occurrence across different health regions of the country. The epidemiological landscape of SARS has been influenced by population density, healthcare infrastructure, and public health interventions (Cardoso et al., 2019; Oliveira et al., 2009; Ribeiro and Sanchez, 2020; Trombetta et al., 2016). Tracking the prevalence and incidence provides essential insights into SARS incidence's geographical and temporal patterns. This information is critical for understanding the impact of the disease on communities, guiding public health strategies, and evaluating the effectiveness of control measures implemented.

Numerous studies have been sought to model the impact of both SARS and COVID-19 cases and deaths worldwide and also in Brazil. These were done in temporal, spatial, and spatiotemporal perspectives. For example, Cao et al. (2010) presented an empirical spatio-temporal analysis of epidemiological data concerning 2321 SARS-infected patients in Beijing in 2003, Pereira et al. (2021) consider both survival analysis and singular spectrum analysis to forecast the number of deaths in Brazil and in Italy, Oliveira et al. (2021) used a mathematical model to study the dynamics of COVID-19 in Bahia, a state in northeastern Brazil, and Siljander et al. (2022) used a spatiotemporal clustering method to find patterns and sociodemographic determinants of COVID-19 infections in Helsinki, Finland.

Various studies have also aimed to identify factors or covariates that might influence the incidence and mortality rates of SARS and COVID-19. For instance, Ravelli y Martinez (2022) explored the correlation between specific humidity and influenza/SARS-CoV-2 in the Netherlands, examining temporal and regional patterns. In a different context, Roper y Rehm (2009) conducted a comprehensive review of vaccine development against human SARS. Additionally, both Moghadas et al. (2021) and Victora et al. (2021) analyzed the effects of vaccination against COVID-19 in the United States and Brazil, respectively.

**Fig. 1:**
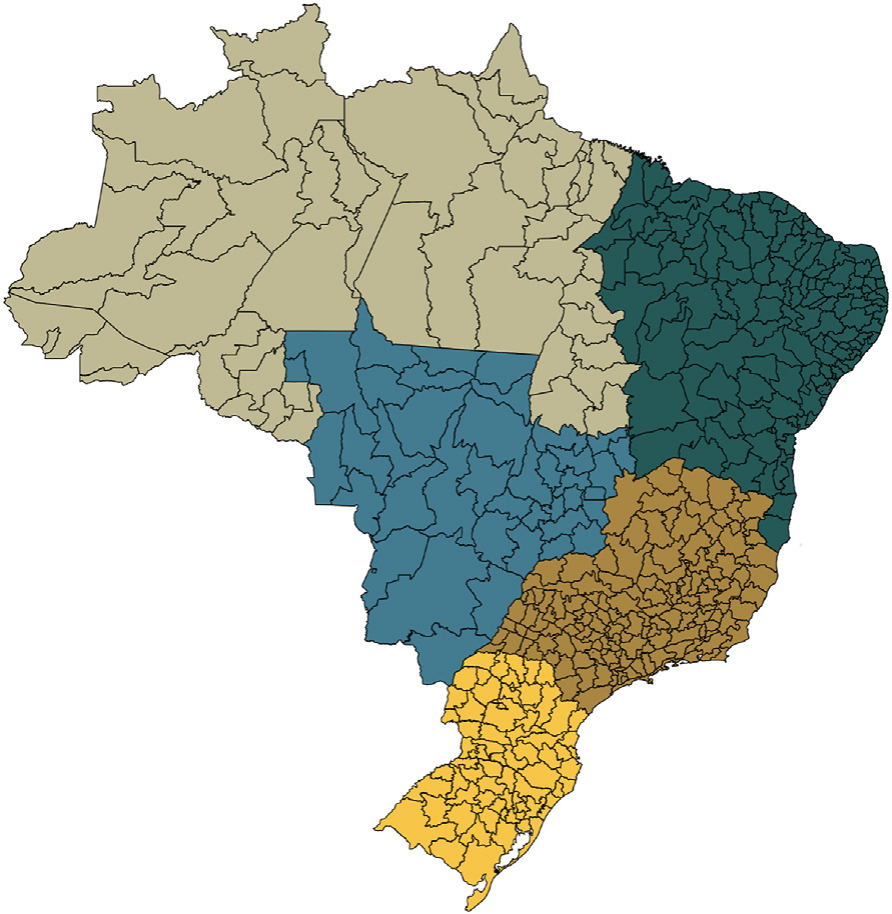
Map of Brazil subdivided into the 438 health regions. The colors denote the five geographic regions of the country.

However, as far as our knowledge extends, no specific study compares SARS cases across the pre-, during, and post-COVID-19 eras. This paper addresses this gap by investigating the spatio-temporal dynamics of SARS cases in Brazil from January 2016 to August 2024, spanning the pre-, during, and post-COVID-19 periods. This comparison allows for a better understanding of the impact of COVID-19 and related public health measures on SARS cases, allowing for useful insights into changes in SARS dynamics before, during, and after the COVID-19 pandemic. The temporal segmentation facilitates a nuanced examination of SARS trends across these eras within the health regions of Brazil (Fig. 1). Following descriptive and exploratory analyses, a spatio-temporal generalized linear model for areal unit data incorporating Bayesian inference for parameter estimation (Lee et al., 2018) is used to model and compare the pre-, during, and post-COVID-19 periods. Climate and vaccination data are used as covariates of the spatio-temporal model. The choice for the use of this model was because it allows (i) to have count data for areal unit data as response variable; (ii) the response variable to vary in space and time; and (ii) to consider covariates.

The remainder of this paper is organized as follows. Section 2 offers a detailed overview of the processes involved in data collection, treatment, and organization. Additionally, we describe the spatio-temporal generalized linear model designed for areal unit data. Section 3 unveils the findings and a discussion, encompassing descriptive and exploratory analyses and the outcomes derived from the spatio-temporal modeling. The paper concludes in Section 4, where we present a final discussion with some concluding remarks and future work directions.

## 2 Materials and methods

This section describes the data utilized in this analysis, covering its collection, treatment, and organization processes for both the response variable and the covariates. Subsequently, we delve into the specifics of the spatio-temporal generalized linear model designed for areal unit data, whose inferences about its parameters are made in a Bayesian framework.

### 2.1 The data

Our study considers complex data spanning approximately eight years from January 3, 2016, to August 31, 2024. The data includes information from three distinct sources.

(i) records of severe acute respiratory syndrome (SARS) cases across the Brazilian territory sourced from DATASUS, the health information system maintained by the Brazilian Ministry of Health (datasus.saude.gov.br);
(ii) hourly climate data gathered from all available meteorological stations in Brazil, provided by the Brazilian National Institute of Meteorology (INMET; Instituto Nacional de Meteorologia; portal. inmet.gov.br);
(iii) vaccination records against COVID-19, also obtained from DATASUS.

The data selected for modeling is organized spatially by health region, with health regions established as organizational units within the Unified Health System (SUS; Sistema Único de Saúde) in Brazil. A group of neighboring municipalities forms a health region. These health regions (Fig. 1) facilitate the management and integration of health actions and services at regional levels, their demarcation guided by demographic, epidemiological, socioeconomic, and geographic criteria. Brazil has a total of 456 health regions. This number is updated over the years, and municipalities may be included in new health regions, or new regions may be created. Despite 456 existing regions, only 438 health regions will be used in this study (Fig. 1). This difference is due to the need to use a spatial mesh to determine the matrix of neighborhoods among the regions, the most recent of which was made available in 2013 when there were 438 health regions. Temporally, the data is aggregated by epidemiological week, a standard unit of measurement in epidemiology used in Brazil for monitoring diseases such as influenza, dengue, zika, and chikungunya (PAHO, 2016).

The study period is defined based on epidemiological weeks, commencing on January 3, 2016, with the initiation of the first epidemiological week of 2016 and concluding on August 31, 2024. Three distinct time segments were established for the modeling phase to formulate three models: pre-COVID-19, during, and post-COVID-19. The cutoff points for these periods were determined as the date of the World Health Organization's (WHO) declaration of the health emergency caused by COVID-19 (2020-03-11) and the date of the WHO's announcement of the end of the health emergency caused by COVID-19 (2023-05-05).

However, the periods are defined by the commencement and conclusion of epidemiological weeks due to slight variations in the two dates. Specifically, the pre-COVID-19 period spans from epidemiological week 01 of 2016 (2016-01-03) to epidemiological week 11 of 2020, concluding on 2020-03-14, three days after the WHO's pandemic declaration, thus resulting in 219 epidemiological weeks. The COVID-19 period spans from epidemiological week 11 of 2020 to epidemiological week 18 of 2023, ending on 2023-05-06, one day after the WHO announced the pandemic's end, thus resulting in 164 epidemiological weeks. The post-COVID-19 period extends from epidemiological week 18 of 2023 to epidemiological week 35 of 2023 (2023-08-31), thus resulting in 69 epidemiological weeks.

**Fig. 2:**
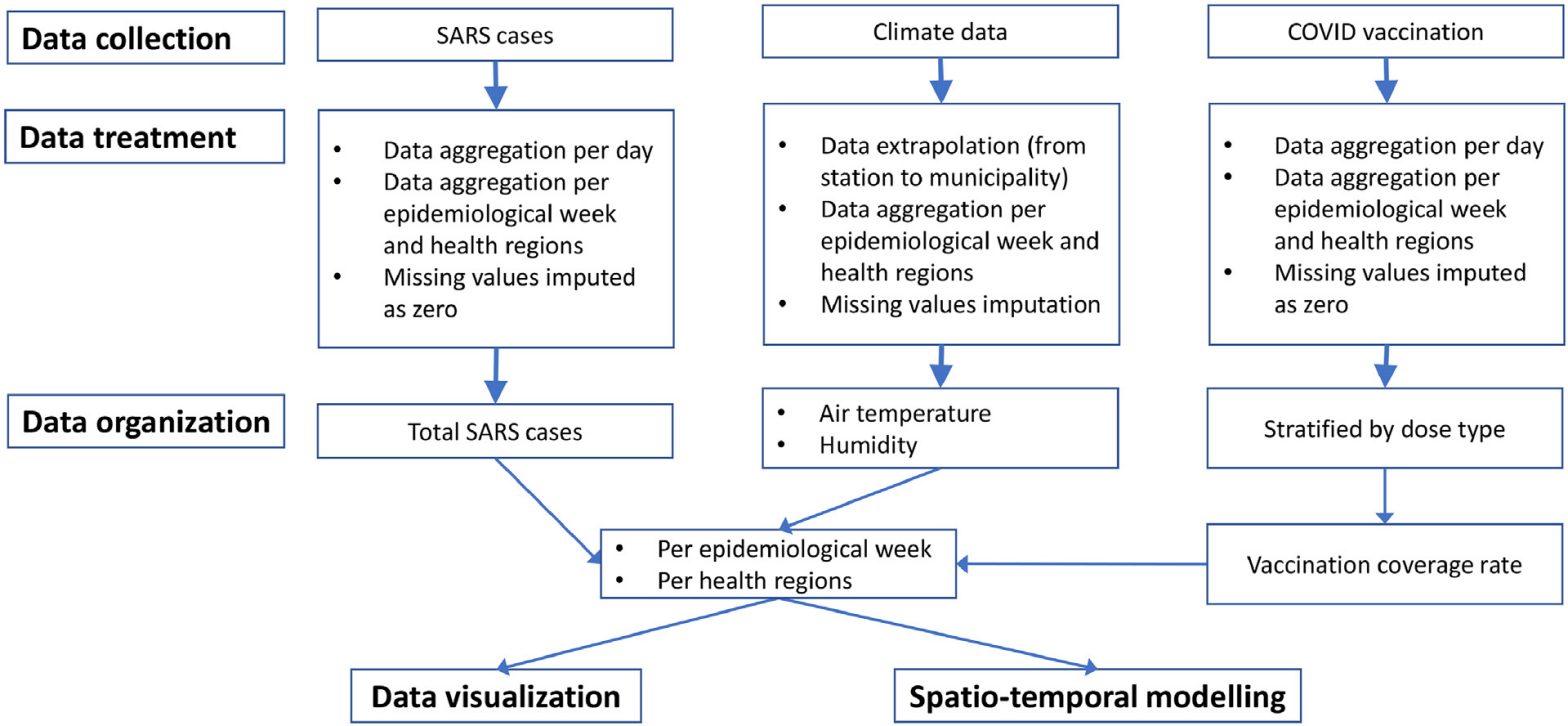
Detailed flowchart illustrating the methodology employed in this paper, encompassing data collection, treatment, organization, visualization, and spatial-temporal modeling.

Considering the diverse formats and sources of the data, various approaches were employed to transform it into the final format required for analysis. A comprehensive flowchart illustrating the proposed methodology is presented in Fig. 2. Detailed information on data collection, processing, and organization is provided below to facilitate reproducibility in scientific endeavors.

The response variable, total SARS cases, initially spanned eight separate files–one for each year–encompassing all registered SARS cases in Brazilian territory. These raw files contained over 130 variables, including case notification date, patient age, and notification location. Over the eight-year study period, more than 3.8 million SARS cases were documented, corresponding to the number of lines in the associated database. To streamline the dataset, cases reported over the eight years were initially aggregated based on the municipality and reporting day. Subsequently, these aggregate cases were grouped based on epidemiological weeks and the health region associated with the notifying municipality. This process resulted in a database with 404 rows and 438 columns, where each row represents an epidemiological week, and each column represents a health region. We assume that a zero value was assigned for weeks where no observations were recorded in a particular health region, signifying the absence of reported cases during that specific week in that location.

Regarding climate data, the original dataset comprises a file for each weather station, and the number of stations increases over time, starting with 529 in 2016 and reaching 566 in 2023 (529, 563, 596, 589, 588, 567, and 566 for the years between 2016 and 2023, respectively). These files contain information such as date, time, total precipitation, atmospheric pressure (hourly, maximum, and minimum), global radiation, air temperature, dew point temperature (hourly, maximum, and minimum), hourly temperature (minimum and maximum), humidity (hourly, minimum, and maximum), and wind (direction, maximum gust, and hourly speed). The selected variables for analysis were air temperature and humidity, measured in degrees Celsius (°C) and percentage (%), respectively. Each file contains 8760 lines for “typical” years and 8784 for leap years. Since the original data was collected hourly, weekly averages were calculated for each weather station. Due to the uneven distribution of meteorological stations across Brazilian municipalities and a notable number of missing observations, the initial extrapolation for each municipality involved calculating a weighted average from the three meteorological stations closest to the municipality that had observations (Pimentel et al., 2024). This was done after evaluating the six closest weather stations, with greater weight assigned to stations closer to the municipality.

The last database used, about COVID-19 vaccination, posed the greatest challenge due to its size. The original data comprised 20 extensive databases encompassing all records of COVID-19 vaccines administered in Brazil up to the collection date. These raw files contained 32 variables, including vaccination location, age and sex of the vaccinated person, type of vaccine, and more. Commencing in January 2021, the data reflects the total doses administered between 2021 and 2023, encompassing the first and second doses and the first and second booster doses. This resulted in a dataset with more than 540 million doses administered, corresponding to the same number of rows. For this dataset, the number of vaccines was initially aggregated based on the location and day of administration. Following this initial aggregation, the doses were further grouped based on epidemiological week and health region associated with the administering municipality. Stratification was also performed based on the type of dose (1st dose, 2nd dose, 1st booster dose, and 2nd booster dose), resulting in four distinct databases with 404 lines (i.e., the number of epidemiological weeks) and 438 columns (i.e., the health region) each.

In this way, the data to be used for analysis and modeling are composed of the response variable with the total number SARS cases, the climatic covariates air temperature and humidity, and covariates on vaccination against COVID-19 with the first two doses and the first two booster doses.

All analyses were conducted using the R programming language (version 4.3.2) (R Core Team, 2023) on a personal computer equipped with a 2.50 GHz Intel Core i5-10300H processor and 8 GB RAM, running the Windows 11 64-bit operating system.

### 2.2 Bayesian spatio-temporal model

Conventional linear regression models assume independent observations and neglect temporal and spatial dependencies. Given the nature of our panel data, which varies across both dimensions, it is crucial to account for these dependencies. To address this, we have employed a spatio-temporal generalized linear model tailored for areal unit data. In this model, parameter inferences are made within a Bayesian framework, enabling us to capture the inherent dependencies over time and space present in our dataset more effectively. These models are well-suited for fitting areal unit data observed in discrete periods while accommodating the inclusion of pertinent explanatory variables. As proposed by Lee et al. (2018), the model is specified as follows:

$$\begin{array}{lll} Y_{k,t}|\mu_{k,t} & ∼ & Poisson\left(o_{k,t}\mu_{k,t}\right),\\ \ln\mu_{k,t} & = & x_{k,t}^{⊺}\beta +\psi_{k,t},\\ \beta & ∼ & N\left(\mu_{\beta },\Sigma_{\beta }\right),\\ \psi_{k,t} & = & \beta_{0}+\phi_{k}+\left(\alpha +\delta_{k}\right)\frac{t-\bar{t}}{N},\\ \phi_{k}|\phi_{-k},W & ∼ & N\left(\mu_{\phi_{k}|\phi_{-k},W},\sigma_{\phi_{k}|\phi_{-k},W}^{2}\right),\\ \delta_{k}|\delta_{-k},W & ∼ & N\left(\mu_{\delta_{k}|\delta_{-k},W},\sigma_{\delta_{k}|\delta_{-k},W}^{2}\right),\\ \tau_{\text{int}}^{2},\tau_{\text{slo}}^{2} & ∼ & \text{Inverse}-\text{Gamma}\left(a,b\right),\\ \rho_{\text{int}},\rho_{\text{slo}} & ∼ & Uniform\left(0,1\right),\\ \alpha & ∼ & N\left(\mu_{\alpha },\sigma_{\alpha }^{2}\right), \end{array}$$

where.

• E[*Y*_*k*,*t*_∣*μ*_*k*,*t*_] = *o*_*k*,*t*_*μ*_*k*,*t*_ denotes the conditional expectation of *Y*_*k*,*t*_∣*μ*_*k*,*t*_, where *μ*_*k*,*t*_ is the risk of SRAG in health region *k* during month *t* relative to an offset *o*_*k*,*t*_;
• *ψ*_*k*,*t*_ is a latent component for municipality *k* and month *t* embracing one or more sets of spatiotemporally autocorrelated random effects;
• ***β*** = (*β*_0_, *β*_1_, …, *β*_*p*−1_) is a *p*-dimensional vector of covariate regression parameters;
• **W** = [*w*_*k*,*j*_] is a binary neighborhood K × K matrix, with *w*_*k*,*j*_ = 0 if *k* = *j* (diagonal elements equal to zero), *w*_*k*,*j*_ = 1 if the municipalities *k* and *j* share a common border, and *w*_*k*,*j*_ = 0 if the municipalities *k* and *j* do not share a common border;
• The random effects ***ϕ*** = (*ϕ*_1_, …, *ϕ*_*K*_) and ***δ*** = (*δ*_1_, …, *δ*_*K*_) are modeled as spatially autocorrelated by the CAR prior, satisfying $\overset{K}{\underset{k=1}{\sum }}\phi_{k}=\overset{K}{\underset{k=1}{\sum }}\delta_{k}=0$, with ***ϕ***_−*k*_ and ***δ***_−*k*_ denoting the vectors ***ϕ*** and ***δ*** without their corresponding *k*-th entries, respectively;
• *ρ*_int_ and *ρ*_slo_ are two spatial dependence parameters;
• The quantities $\mu_{\phi_{k}|\phi_{-k},W}$, $\sigma_{\phi_{k}|\phi_{-k},W}^{2}$, $\mu_{\delta_{k}|\delta_{-k},W}$ and $\sigma_{\delta_{k}|\delta_{-k},W}^{2}$ are given by:

$$\mu_{\phi_{k}|\phi_{-k},W}=\frac{\rho_{\text{int}}\overset{K}{\underset{j=1}{\sum }}w_{k,j}\phi_{j}}{\rho_{\text{int}}\overset{K}{\underset{j=1}{\sum }}w_{k,j}+1-\rho_{\text{int}}},$$

$$\sigma_{\phi_{k}|\phi_{-k},W}^{2}=\frac{\tau_{\text{int}}^{2}}{\rho_{\text{int}}\overset{K}{\underset{j=1}{\sum }}w_{k,j}+1-\rho_{\text{int}}},$$

$$\mu_{\delta_{k}|\delta_{-k},W}=\frac{\rho_{\text{slo}}\overset{K}{\underset{j=1}{\sum }}w_{k,j}\delta_{j}}{\rho_{\text{slo}}\overset{K}{\underset{j=1}{\sum }}w_{k,j}+1-\rho_{\text{slo}}},$$

and

$$\sigma_{\delta_{k}|\delta_{-k},W}^{2}=\frac{\tau_{\text{slo}}^{2}}{\rho_{\text{slo}}\overset{K}{\underset{j=1}{\sum }}w_{k,j}+1-\rho_{\text{slo}}}.$$

The model captures the spatio-temporal pattern in the mean response by incorporating a spatially varying linear time trend. The *k*-th health region has its individual linear time trend characterized by a spatially varying intercept *β*_0_ + *ϕ*_*k*_ and a spatially varying slope *α* + *δ*_*k*_. The hyperparameters ***μ***_***β***_, **Σ**_***β***_, *a*, *b*, *μ*_*α*_, and $\sigma_{\alpha }^{2}$ are chosen to have non-informative prior distributions. In this work, we consider the offset *o*_*k*,*t*_ as the population size in the *k*-th health region, which is sent to the model on the log scale.

The spatio-temporal modeling aims to model the total number of SARS cases in Brazil for a given period, aggregated by health region and epidemiologic week. Our consideration of explanatory variables includes humidity, temperature, and the percentages/coverage of the first four doses of the COVID-19 vaccine in the respective health region.

## 3 Results

### 3.1 Spatial and temporal data visualization

**Fig. 3:**
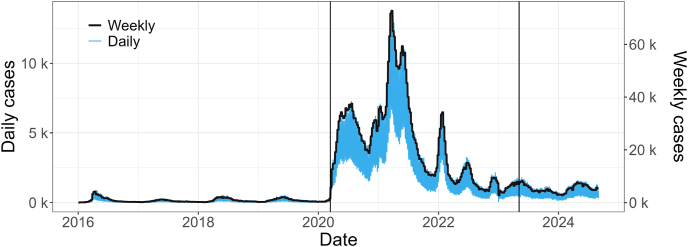
Total SARS cases per day and epidemiological week from January 3, 2016, to August 31, 2024. The vertical black lines denote the cutoff points for the three formulated models: pre-COVID-19 (2016–2020), COVID-19 (2020–2023), and post-COVID-19 (2023–2024).

Our objective with this study is to compare the behavior of SARS cases between the periods before, during, and after the COVID-19 pandemic. With this in mind, when observing the behavior of the number of SARS cases within the study period that goes from January 3, 2016, to August 31, 2024, see Fig. 3 which shows the total SARS cases per day and epidemiological week, considering the three periods under study, the large increase in cases during the period that included the COVID-19 pandemic is notable. The same behavior can be observed when evaluating the behavior of these cases for epidemiological weeks throughout the health regions divided by geographic regions present in Fig. S1 of the supplementary material, where we have the boxplots per epidemiological week considering all health regions.

**Fig. 4:**
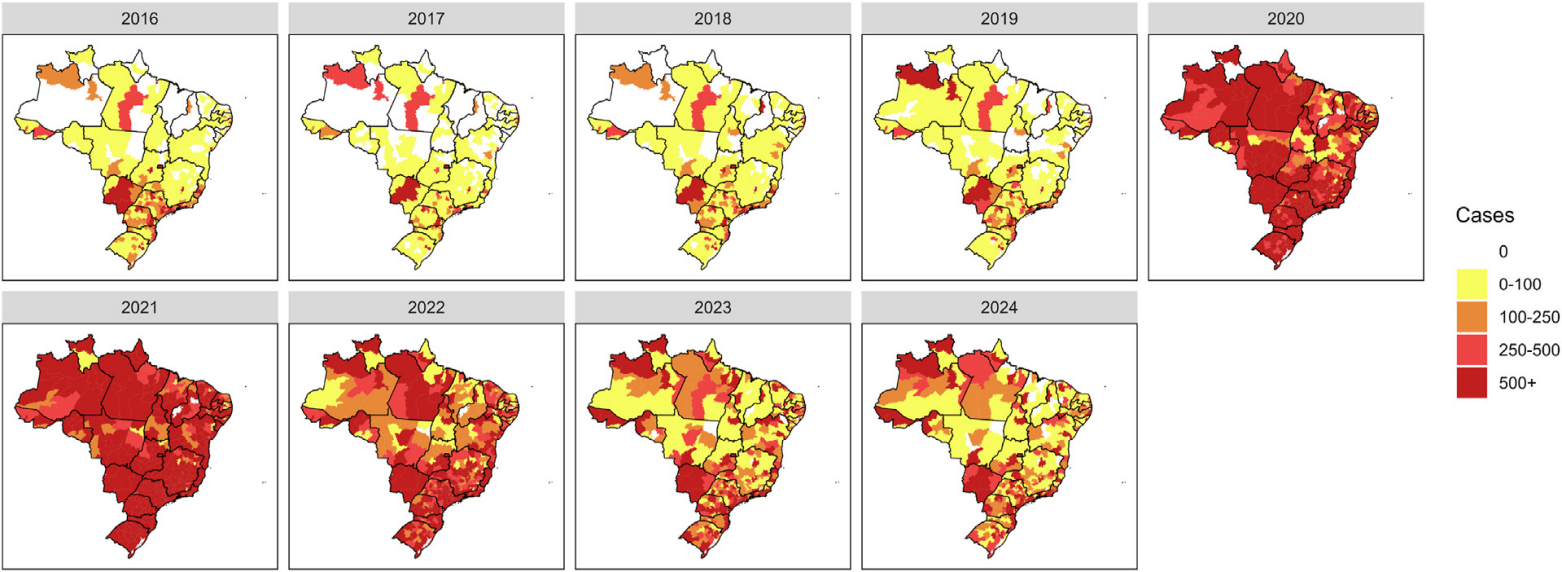
Heat-maps with the evolution in the total number of cases of Severe Acute Respiratory Syndrome (SARS) across all 438 health regions of Brazil from 2016 to 2024.

The same behavior can be observed from the set of heat maps present in Fig. 4, which presents the evolution in the total number of cases of Severe Acute Respiratory Syndrome (SARS) across all 438 health regions of Brazil from 2016 to 2024. It is possible to observe the years 2020 and 2021 as the worst during the study period, where an increase in SARS cases was also observed in addition to the existence of a pandemic. During 2022, despite still high numbers of SARS cases, it is already possible to observe an improvement in the situation. In the years 2023 and 2024, when we have the end of the COVID-19 pandemic, despite control of the situation, it is possible to observe a new disease behavior, with a situation slightly worse than that observed before the pandemic.

Complementary to this analysis, Fig. S2 and S3 in the supplementary material depict the spatial distribution through heatmaps and the statistical summaries using boxplots, respectively, of the mean number of SARS cases per week during the pre-COVID-19, COVID-19, and post-COVID-19 periods.

These visualizations highlight the pronounced increase in SARS cases during the COVID-19 pandemic, with peaks reaching up to 2000 cases per epidemiological week, contrasting to the pre-COVID-19 era, where the maximum was limited to 80 cases. Additionally, comparing the situations before and after the pandemic reveals a notable shift in the “new normal,” characterized by a sustained increase in the average number of cases in some health regions. This increase, averaging up to 400 cases per epidemiological week, underscores the enduring impact and challenges posed by the circulation of new viruses even after the pandemic has been controlled.

The spatio-temporal behavior of the covariates used in this study can be found in Figs. S4–S9 of the supplementary material. The commutative coverage of the four first COVID-19 doses of vaccination every three months during the COVID-19 pandemic across all health regions of Brazil is shown in Figs. S4–S7. Comparing the behavior of the four figures, it is possible to notice the difference in the speed of vaccination progress. Examining the initiation of vaccination, as depicted in Fig. S4, a slower pace is evident initially, attributable to the limited availability of vaccine doses. Notably, especially for the first two doses, a plateau in the vaccination rate becomes apparent from March 2022 onward. This indicates a saturation point in vaccination uptake, possibly reflecting reduced public interest in seeking vaccines after achieving a high level of coverage. As for the booster vaccines, as illustrated in Fig. S6 and S7, it is discernible that the attained coverage is considerably lower compared to the initial two vaccine doses. In September 2023, no health regions surpassed a 60% vaccination coverage for booster doses, underscoring a noteworthy gap in achieving higher levels of coverage for this category of vaccinations.

Regarding climatic variables, Fig. S9 and S10 illustrate the cyclical behavior of air humidity and temperature, respectively. These figures indicate a consistent cyclic pattern without observable variations between the distinct periods evaluated and modeled in the study.

### 3.2 Spatio-temporal modeling

Based on the data visualization results, we observed that the number of SARS cases exhibits dependencies on both time and space. Furthermore, the incidence of SARS cases varies significantly over time, particularly in the pre-COVID-19, during COVID-19, and post-COVID-19 eras. Recognizing this temporal heterogeneity, we tailored a spatio-temporal model for each era to capture the epidemiological weekly totals of cases in each health region rather than proposing a single model for the entire period. In this context, we employ a Bayesian spatio-temporal generalized linear model proposed by Lee et al. (2018), chosen for its suitability in handling count data and enabling the inclusion of explanatory variables.

We worked with the *N* = 438 Brazilian health regions and considered 219, 164, and 69 epidemiological weeks for each of the three eras, respectively. We consider the correlation strength between each candidate variable and the response variable to select explanatory variables in each model.

Table 1, Table 2, Table 3 display the most relevant coefficients of the pre-COVID-19, COVID-19, and post COVID-19 models, respectively. They include the strength of spatial dependence coefficients and the significance of the coefficients associated with the explanatory variables. Due to the Bayesian paradigm adopted in this work, we consider that each component of ***β*** is statistically significant when zero is outside the credibility interval formed by the 2.5-th and 97.5-th percentiles of its corresponding posterior distribution. Humidity and temperature were significant in the pre-COVID-19 model, while temperature and the four COVID-19 vaccines were significant in the model for the COVID-19 period. The post-COVID-19 model has the same covariates as the pre-COVID-19 model, humidity and temperature. It is crucial to interpret these findings cautiously, recognizing that they do not signify the cessation of COVID-19 virus circulation. The diminishing efficacy of the first four doses of the vaccine over time implies that these explanatory variables may no longer be as effective in elucidating the causes of the most recent cases of SARS.

**Table 1:** Posterior mean (PM), 2.5-th and 97.5-th percentiles of the posterior distribution of the parameters of the pre-pandemic model. The acceptance rate in percentage (AR%), the effective sample size (ESS) and the Geweke statistic (GS) for each model parameter is provided. The sample size of the posterior distribution of each parameter is equal to one thousand.

Parameter	PM	2.5-th perc.	97.5-th perc.	AR%	ESS	GS
*β*_0_	−118.983	−120.424	−117.531	44.9	590.8	1.5
*β*_Humidity_	0.0053	0.0039	0.0067	44.9	622.0	−0.4
*β*_Temperature_	−0.1129	−0.1174	−0.1083	44.9	670.8	−1.4
*α*	0.1288	0.0341	0.2167	36.9	629.2	1.1
$\tau_{\text{int}}^{2}$	82.485	66.762	99.570	100.0	1000.0	−1.5
$\tau_{\text{slo}}^{2}$	57.163	42.856	73.886	100.0	1000.0	0.5
*ρ*_int_	0.8225	0.6578	0.9584	40.3	1000.0	−0.1
*ρ*_slo_	0.6581	0.4210	0.8836	44.1	889.5	0.3

**Table 2:** Posterior mean (PM), 2.5-th and 97.5-th percentiles of the posterior distribution of the parameters of the pandemic model. The acceptance rate in percentage (AR%), the effective sample size (ESS) and the Geweke statistic (GS) for each model parameter is provided. The sample size of the posterior distribution of each parameter is equal to one thousand.

Parameter	PM	2.5-th perc.	97.5-th perc.	AR%	ESS	GS
*β*_0_	−76.124	−76.325	−75.907	47.0	170.7	−0.7
*β*_Temperature_	−0.0362	−0.0370	−0.0355	47.0	285.7	0.5
*β*_1st shot_	−0.0144	−0.0147	−0.0142	47.0	56.6	0.2
*β*_1st booster shot_	−0.0096	−0.0100	−0.0093	47.0	49.5	−0.3
*β*_2nd shot_	−0.0075	−0.0078	−0.0071	47.0	43.4	0.2
*β*_2nd booster shot_	−0.0578	−0.0583	−0.0572	47.0	245.2	1.0
*α*	25.734	25.505	25.974	33.7	141.4	−0.9
$\tau_{\text{int}}^{2}$	25.504	20.948	30.951	100.0	1000.0	0.0
$\tau_{\text{slo}}^{2}$	55.115	45.713	65.804	100.0	1000.0	−1.4
*ρ*_int_	0.7532	0.5812	0.9266	49.2	1000.0	1.0
*ρ*_slo_	0.7955	0.6193	0.9411	45.3	1000.0	−0.5

**Table 3:** Posterior mean (PM), 2.5-th and 97.5-th percentiles of the posterior distribution of the parameters of the post-pandemic model. The acceptance rate in percentage (AR%), the effective sample size (ESS) and the Geweke statistic (GS) for each model parameter is provided. The sample size of the posterior distribution of each parameter is equal to one thousand.

Parameter	PM	2.5-th perc.	97.5-th perc.	AR%	ESS	GS
*β*_0_	−114.976	−115.711	−114.208	46.2	1000.0	−0.4
*β*_Humidity_	0.0060	0.0052	0.0068	46.2	1000.0	0.8
*β*_Temperature_	−0.0321	−0.0342	−0.0300	46.2	1000.0	−0.2
*α*	0.0408	−0.0084	0.0939	34.0	1000.0	0.3
$\tau_{\text{int}}^{2}$	53.564	40.137	70.709	100.0	1000.0	−0.9
$\tau_{\text{slo}}^{2}$	19.143	15.701	24.315	100.0	1000.0	−0.1
*ρ*_int_	0.4212	0.2499	0.6317	40.0	1000.0	−0.1
*ρ*_slo_	0.0173	0.0005	0.0669	51.7	1000.0	−1.4

**Fig. 5:**
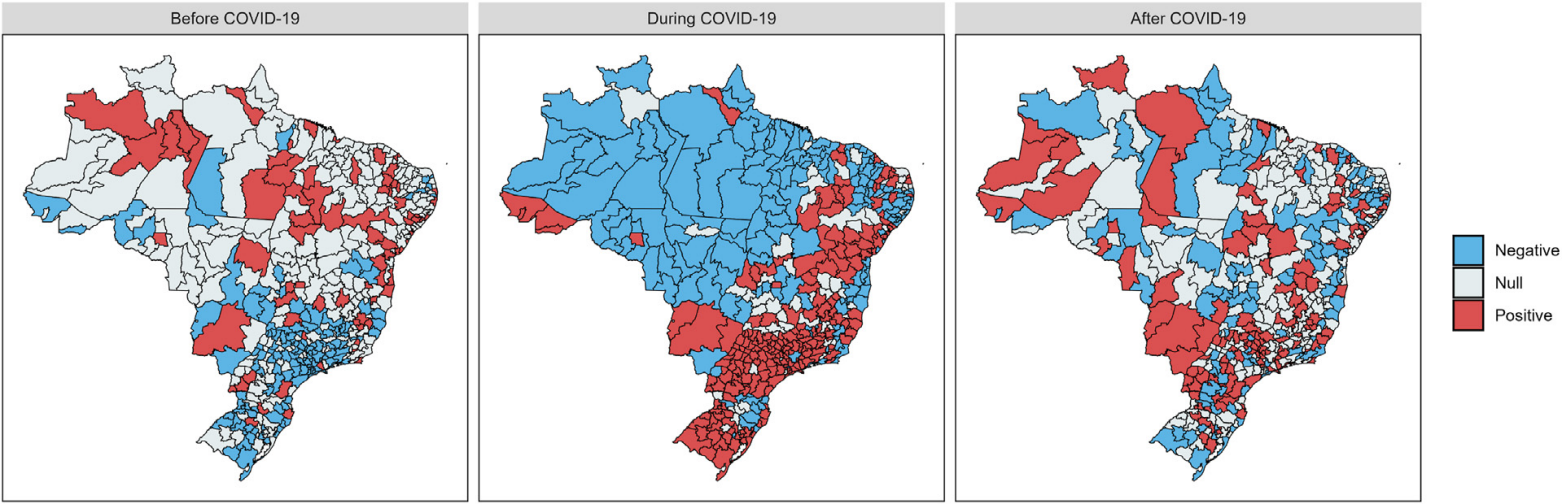
Heat maps with the sign of the incremental slope parameter *δ*_*k*_ relative to the *k*-th Brazilian health regions by periods pre-COVID-19, during, and post-COVID-19, being null if zero is contained within its 95% credible interval. The plots were generated with the R software (R Core Team, 2023).

The interaction between the time effect and the area effect is given by the term *δ*_*k*_ (Galarza et al., 2024), which is known as the “differential trend of the *k*-th areal unit”. According to Bernardinelli et al. (1995), we interpret *δ*_*k*_ as follows: a positive value of *δ*_*k*_ implies that the temporal trend of areal unit *k* is steeper than the mean trend *α*, while a negative value of *δ*_*k*_ implies that the temporal trend of the areal unit *k* is less steep than the mean trend *α*. Following Pimentel et al. (2024), we say that *δ*_*k*_ is null if zero is contained within its 95% credible interval. Fig. 5 shows the health regions where *δ*_*k*_ is positive, null, or negative by era (pre-, during, and post-COVID-19), which can be useful for identifying locations that showed variation within the initial and final periods of each model.

**Fig. 6:**
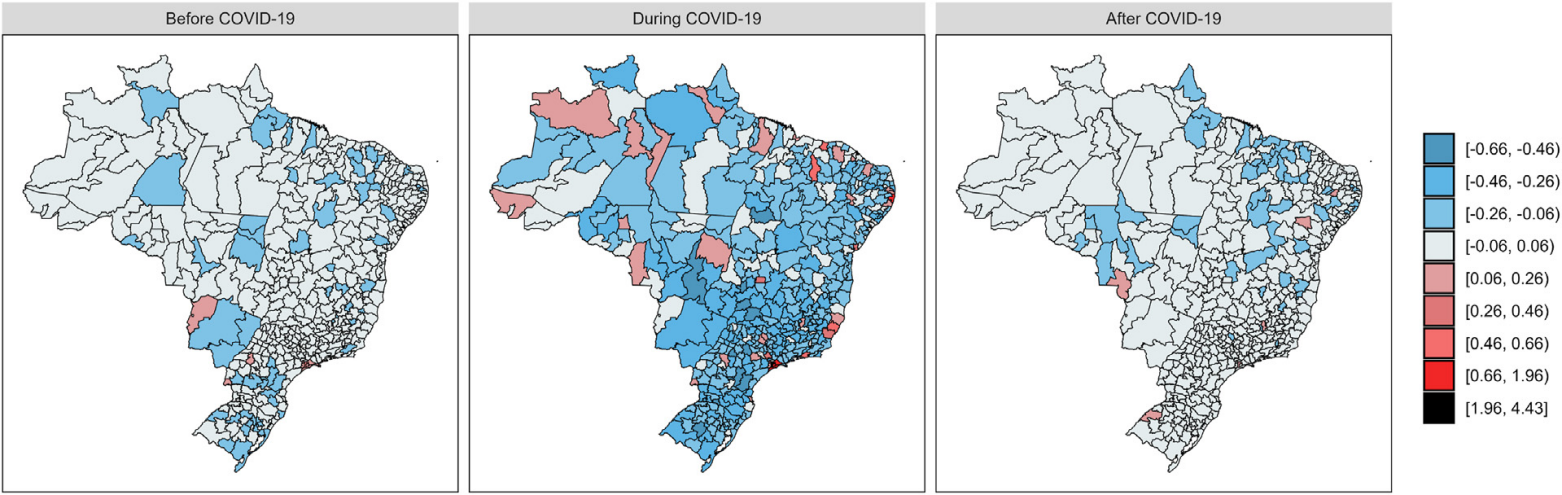
Heat maps with the spatial Pearson residuals for Brazilian health regions by periods pre-COVID-19, during, and post-COVID-19. The plots were generated with the R software (R Core Team, 2023).

**Fig. 7:**
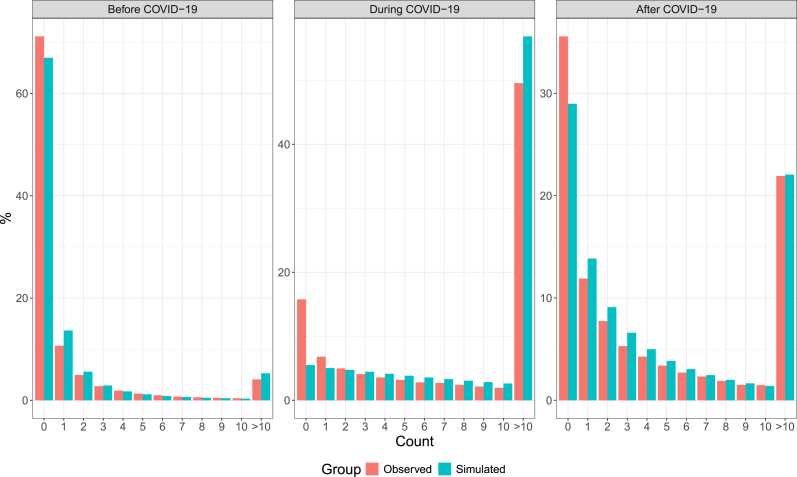
Posterior predictive checking for each Bayesian spatiotemporal model. The plots were generated with the R software (R Core Team, 2023).

Based on Pimentel et al. (2024), we conducted a residual analysis and the Posterior Predictive Checking (PPC) for each model. After obtaining spatial Pearson residuals for each health region *k*, aggregated across all time periods, we will verify if they are inside or outside the interval [−1.96, 1.96]. The spatial Pearson residuals shown in Fig. 6 indicate a good fit for the three models. PPC involves simulating data from the posterior predictive distribution and comparing the simulated data to the observed data. Fig. 7 shows the differences between the relative frequencies of observed data and simulated data in the three models. Since the resulting distributions are similar, it indicates that the models in use are capturing the underlying distribution of the data and are thus considered valid.

## 4 Discussion

In conclusion, our comprehensive Bayesian spatio-temporal modeling of Severe Acute Respiratory Syndrome (SARS) across Brazilian health regions from 2016 to 2023 has provided valuable insights into the multifaceted dynamics of this respiratory syndrome. The study, spanning distinct eras of pre-COVID-19, during-COVID-19, and post-COVID-19, has elucidated the intricate relationships between temporal, spatial, and environmental factors, offering a nuanced understanding of SARS incidence. Considering explanatory variables such as humidity, temperature, and COVID-19 vaccination doses has enhanced the predictive capacity of our models.

Based on initial analyses (Fig. 3, Fig. 4), it was possible to observe the change in behavior in the number of SARS cases during and after the COVID-19 pandemic, where even with control of the new disease, there are signs of a new behavior of the SARS disease with the insertion of yet another virus that causes this disease. Furthermore, when evaluating the covariates used, we noticed cyclical behavior for the climatic variables, without major differences throughout the study period. We also have the behavior observed for the vaccination coverage of the first four doses against COVID-19 applied in the country, where we had a slow pace initially for the first two doses, followed by an acceleration and stabilization at high levels of vaccination coverage, as well as little variation from March 2022 (Berra et al., 2024). For booster doses, a lower demand was observed, with vaccination coverage rates lower than the first two doses, where we have, for example, the second booster dose with coverage of a maximum of 60% in the health regions that most applied this vaccine.

Through statistical modeling, we successfully accommodated count data that vary across epidemiological weeks and health regions for each of the three eras considered in this study. Notably, distinct temporal trend patterns were identified for each health region, revealing spatial relationships resembling neighborhood patterns. Health regions with low (or high) temporal trends often exhibited adjacency to others with corresponding low (or high) temporal trends.

Furthermore, our analysis revealed that low temperature and high humidity were associated with a decrease in SARS cases. These findings underscore the multifaceted interplay of various factors influencing SARS incidence during different eras, providing valuable insights for targeted public health interventions. The effect of temperature and relative humidity in SARS cases have been widely studied, e.g., Chan et al. (2011); Dabisch et al. (2021).

The Bayesian framework employed in our modeling approach has facilitated a robust parameter estimation, capturing the dataset's dependencies and uncertainties Pimentel et al. (2024); Galarza et al. (2024). This enhances the reliability of our findings and provides a methodological contribution to the field of spatio-temporal modeling for respiratory syndromes. However, a limitation of the model is its inability to address zero inflation and overdispersion, which may affect the accuracy of results in regions or periods with few or highly variable cases.

As the global landscape continues to evolve and new challenges in public health emerge, the findings of this study offer a foundation for proactive and data-driven decision-making. Health authorities can tailor interventions, allocate resources effectively, and enhance overall preparedness for respiratory syndromes by understanding the nuanced interplay of factors influencing SARS dynamics. Our research contributes to the ongoing discourse on infectious disease modeling, providing valuable insights for the scientific community, policymakers, and public health practitioners.

Future research directions can include a comparative analysis with other spatiotemporal models and predictive modeling. The examination of different medical and policy practices could also offer practical recommendations for reducing the number of SARS cases. Moreover, it is essential to continue monitoring the spatial and temporal dynamics of SARS cases, particularly in the context of evolving public health conditions and environmental changes. Longitudinal studies are needed to assess the long-term impact of vaccination rates, healthcare resources, and climate factors on SARS incidence and severity.

## CRediT authorship contribution statement

**Rodrigo de Souza Bulhões:** Writing – review & editing, Writing – original draft, Methodology, Investigation, Formal analysis. **Jonatha Sousa Pimentel:** Writing – review & editing, Writing – original draft, Visualization, Software, Methodology, Investigation, Formal analysis, Data curation. **Paulo Canas Rodrigues:** Writing – review & editing, Writing – original draft, Validation, Supervision, Project administration, Investigation, Conceptualization.

## Declaration of competing interest

The authors declare that they have no known competing financial interests or personal relationships that could have appeared to influence the work reported in this paper.
