# Corrigendum to: “Bayesian spatio-temporal modeling of severe acute respiratory syndrome in Brazil: A comparative analysis across pre-, during, and post-COVID-19 eras” [Infectious Disease Modelling, 10 (2) (2025) 466-476]

**DOI:** 10.1016/j.idm.2025.12.017

**Published:** 2025-12-30

**Authors:** Rodrigo de Souza Bulhões, Jonatha Sousa Pimentel, Paulo Canas Rodrigues

**Affiliations:** a Department of Statistics, IME, Federal University of Bahia, Salvador, BA, Brazil; b Department of Statistical Methods, IM, Federal University of Rio de Janeiro, Rio de Janeiro, RJ, Brazil; c Department of Statistics, CCEN, Federal University of Pernambuco, Recife, PE, Brazil; d Econometrics and Business Statistics, Monash University, Australia

In the published version of this article, an error appears in the text describing the association between meteorological variables and reported "SARS" cases. The manuscript states:

"Furthermore, our analysis revealed that low temperature and high humidity were associated with a **decrease** in SARS cases."

However, this statement is inconsistent with the results presented in Tables 1, 2, and 3. The correct interpretation of the results is that **low temperatures and high humidity were associated with an increase, not a decrease, in cases**, reflecting an inverse association with temperature and a positive association with humidity. This error is limited to the textual interpretation and does not affect the statistical analyses, tables, or figures presented in the article.

In addition, the use of the term **"SARS"** may lead to ambiguity, as it is commonly understood to refer to Severe Acute Respiratory Syndrome caused by coronavirus (World Health Organization, 2020). In the context of Brazilian surveillance data, the outcome referred to corresponds to **SRAG** (*Síndrome Respiratória Aguda Grave*), for which the **recommended translation into English is "SARI" (Severe Acute Respiratory Infection) (**Fitzner et al., 2017), not SARS.

Correction of these points would enhance the clarity and scientific accuracy of the article.
