# Supplementary material for: Bayesian spatio-temporal modeling of severe acute respiratory syndrome in Brazil: A comparative analysis across pre-, during, and post-COVID-19 eras
Source: Infect Dis Model. 2024 Dec 19;10(2):466–76. doi: 10.1016/j.idm.2024.12.010 (PMC11743096; doi:10.1016/j.idm.2024.12.010)
Supplement: Multimedia component 1 [file mmc1.pdf]

Bayesian Spatio-temporal Modeling of Severe Acute Respiratory Syndrome in Brazil: A Comparative Analysis across Pre-COVID-19, During, and Post-COVID-19 Eras

Supplementary material

Rodrigo de Souza Bulhões[1,2], Jonatha Sousa Pimentel[3], and Paulo Canas Rodrigues[1,4]

[1]Federal University of Bahia, Brazil
[2]Federal University of Rio de Janeiro, Brazil
[3]Federal University of Pernambuco, Brazil
[4]Monash University, Australia

1
Number of cases of SARS

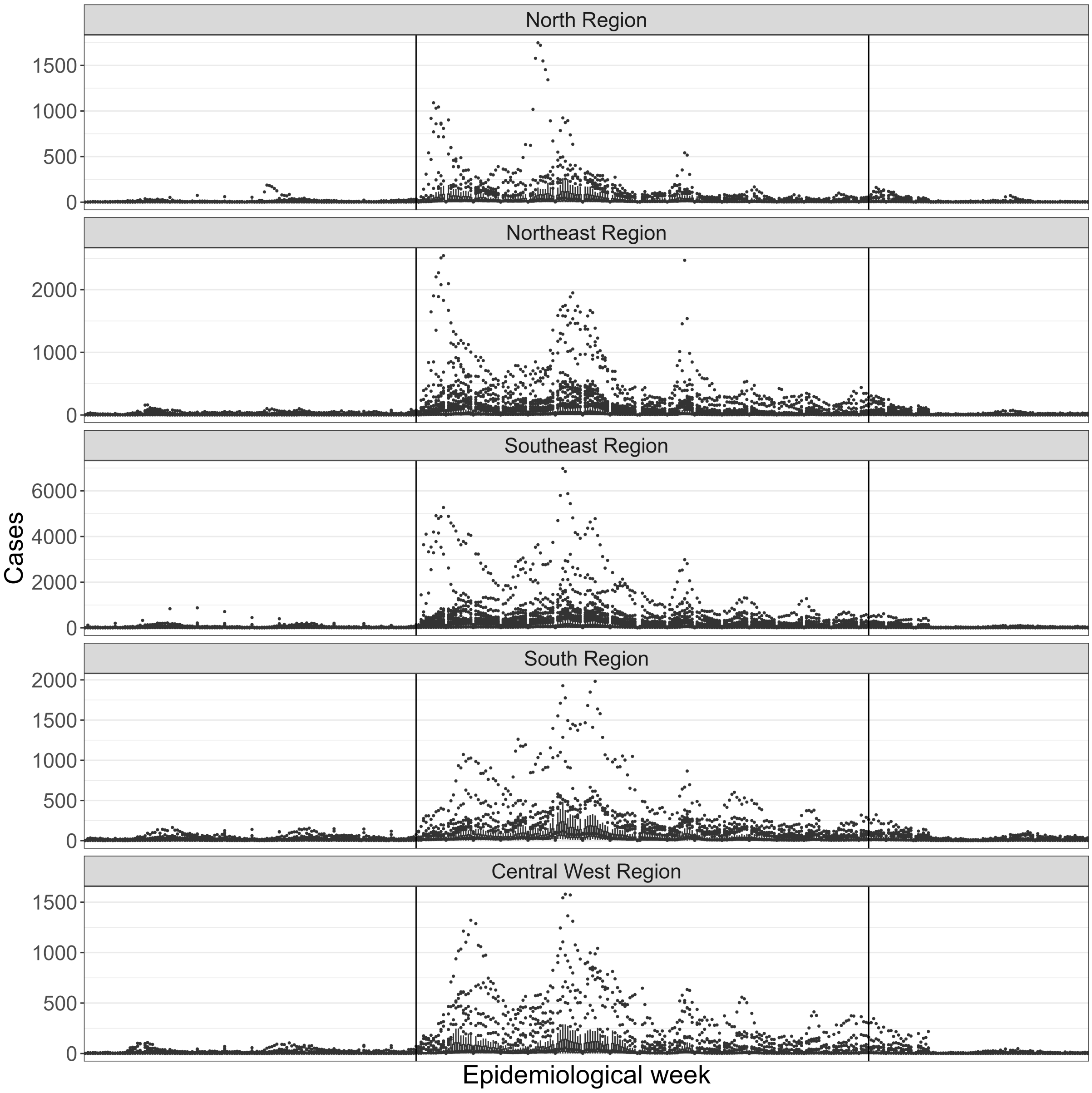

Figure S1: Boxplots for the total number of cases of Severe Acute Respiratory Syndrome (SARS) for all 438 health regions of Brazil along all epidemiological weeks from January 3, 2016, to August 31, 2024.

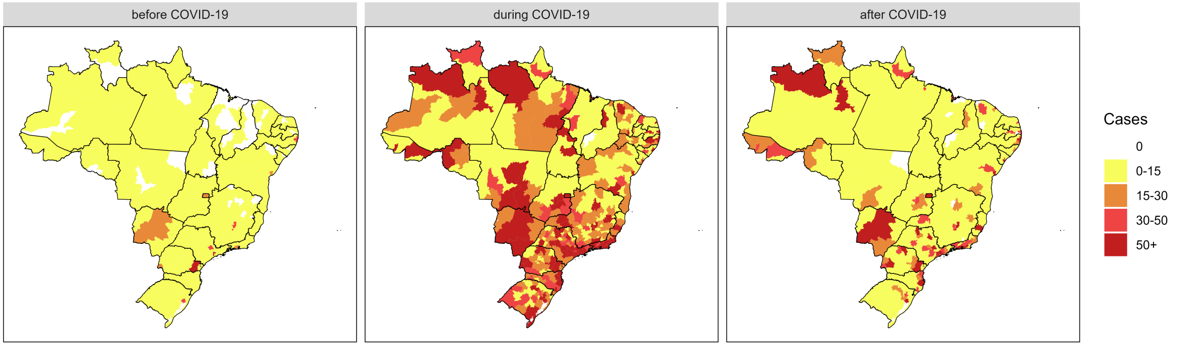

Figure S2: Heat-maps with the mean number of cases of Severe Acute Respiratory Syndrome (SARS) per week in the periods pre-COVID-19, during, and post-COVID-19, across all 438 health regions of Brazil.

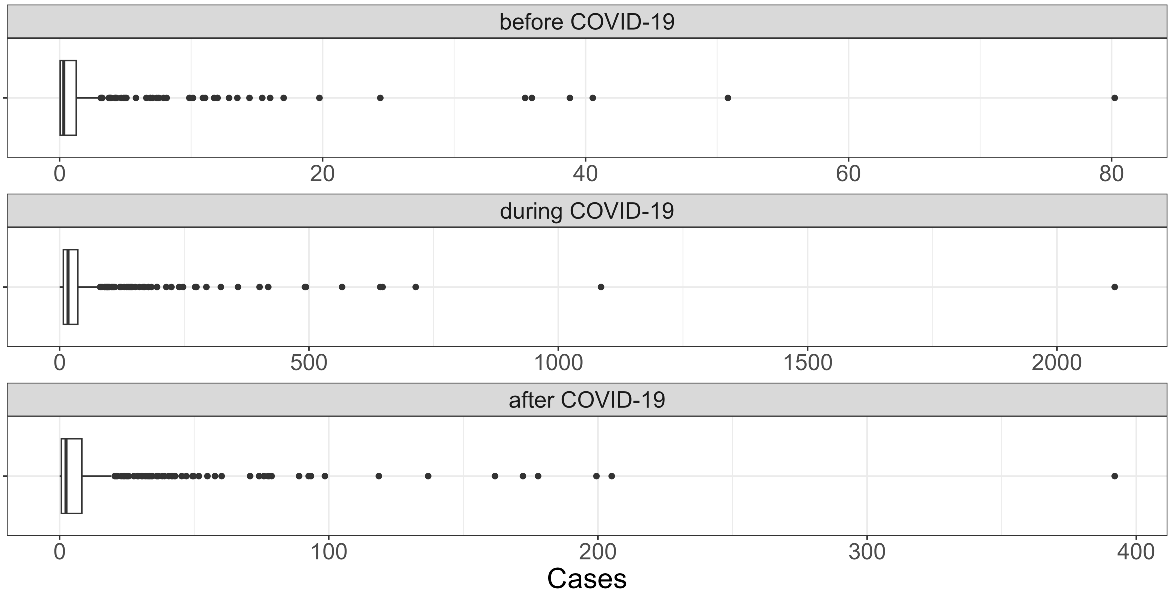

Figure S3: Boxplots for the mean number of cases of Severe Acute Respiratory Syndrome (SARS) per week in the periods pre-COVID-19, during, and post-COVID-19, across all 438 health regions of Brazil.

2
Covariates - Vaccination coverage

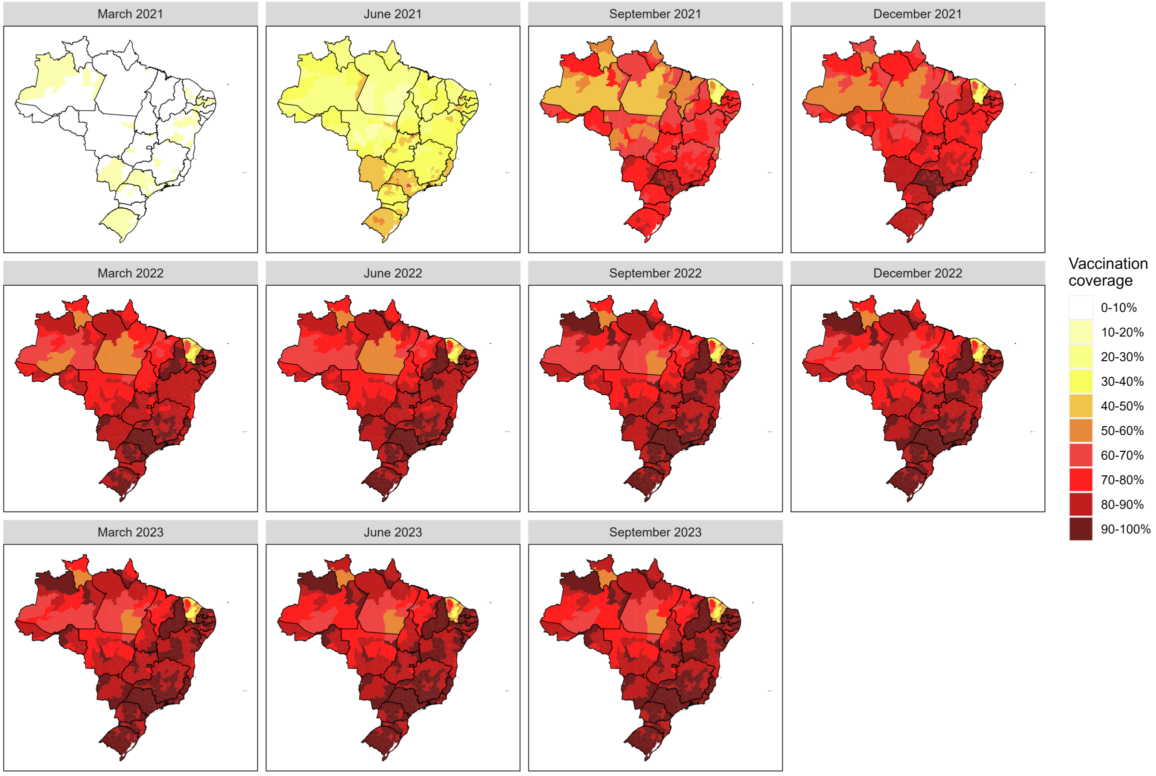

Figure S4: Heat-maps with the vaccine coverage of the first COVID-19 dose every three months during the COVID-19 pandemic, across all 438 health regions of Brazil.

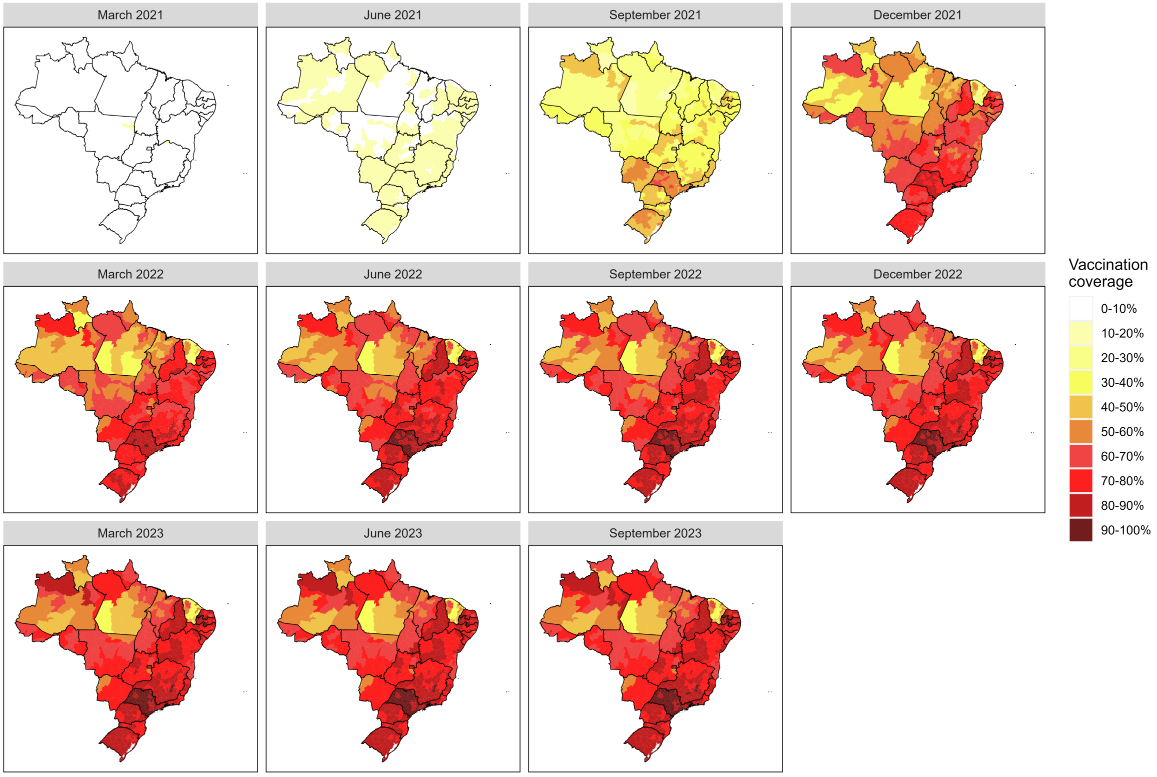

Figure S5: Heat-maps with the vaccine coverage of the second COVID-19 dose every three months during the COVID-19 pandemic, across all 438 health regions of Brazil.

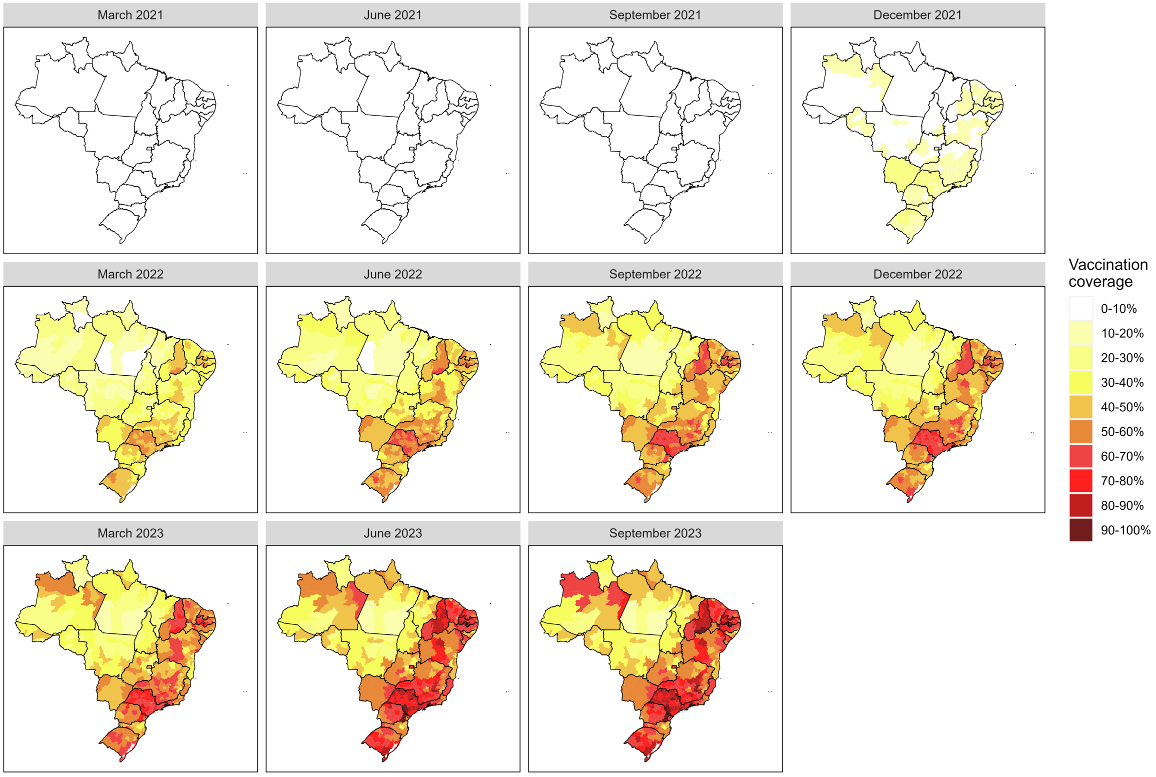

Figure S6: Heat-maps with the vaccine coverage of the first boosted COVID-19 dose every three months during the COVID-19 pandemic, across all 438 health regions of Brazil.

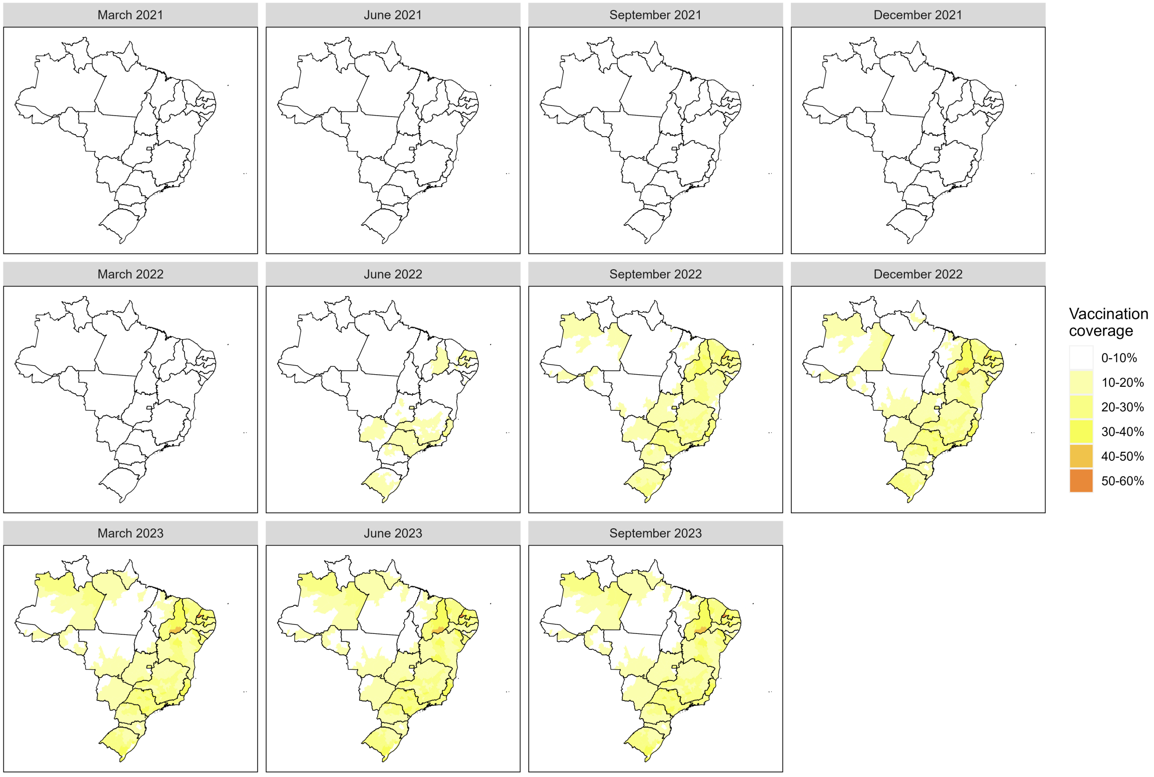

Figure S7: Heat-maps with the vaccine coverage of the second boosted COVID-19 dose every three months during the COVID-19 pandemic, across all 438 health regions of Brazil.

3
Covariates - Climate variables

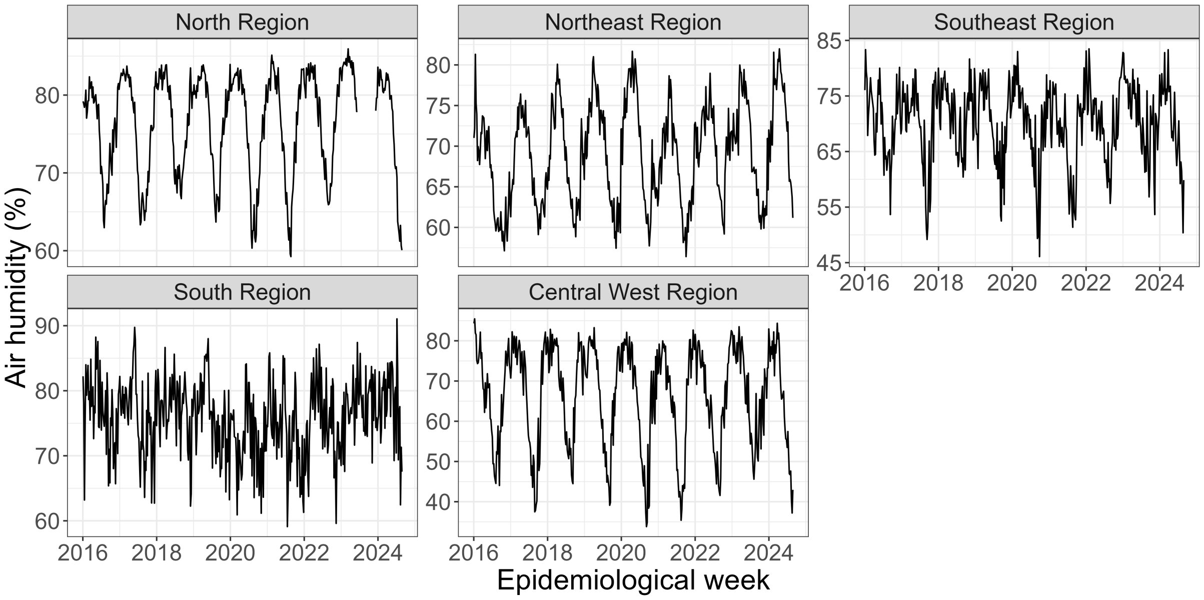

Figure S8: Average air humidity for each geographic region throughout all epidemiological weeks from January 3, 2016, to August 31, 2024.

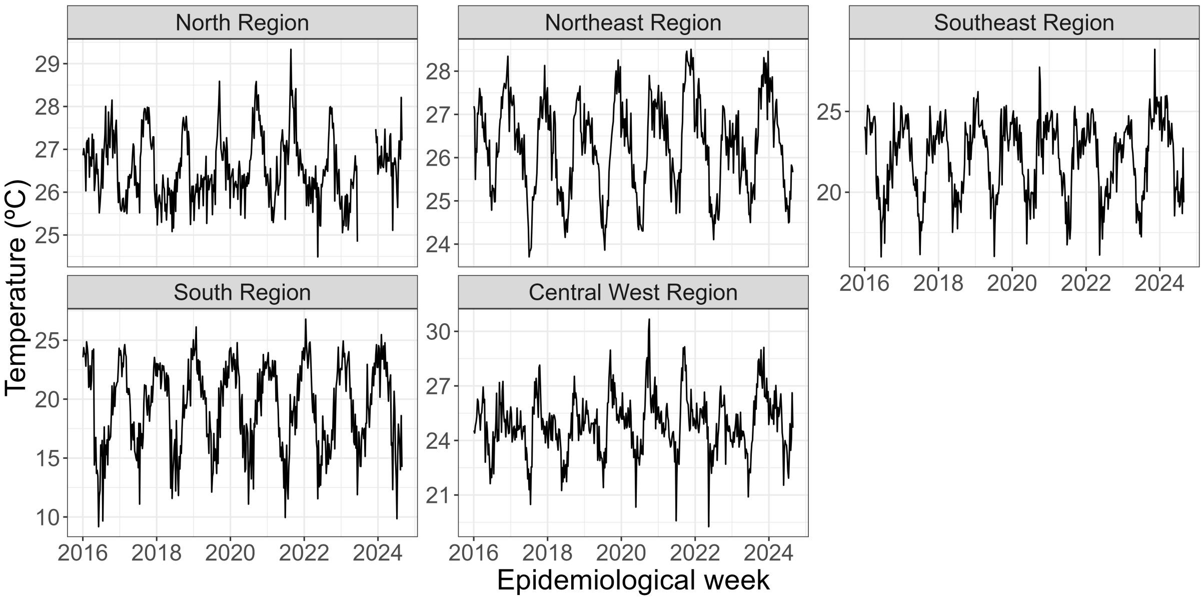

Figure S9: Average temperature for each geographic region throughout all epidemiological weeks from January 3, 2016, to August 31, 2024.
